# Carbon dioxide adsorption and conversion to methane and ethane on hydrogen boride sheets

**DOI:** 10.1038/s42004-022-00739-8

**Published:** 2022-10-04

**Authors:** Taiga Goto, Shin-ichi Ito, Satish Laxman Shinde, Ryota Ishibiki, Yasuyuki Hikita, Iwao Matsuda, Ikutaro Hamada, Hideo Hosono, Takahiro Kondo

**Affiliations:** 1 Graduate School of Pure and Applied Sciences, University of Tsukuba, 1-1-1, Tennodai, Tsukuba, 305-8573 Japan; 2 Materials Research Center for Element Strategy, Tokyo Institute of Technology, Yokohama, 226-8503 Japan; 3 Department of Materials Science and Tsukuba Research Center for Energy Materials Science, Faculty of Pure and Applied Sciences, University of Tsukuba, Tsukuba, 305-8573 Japan; 4 Advanced Research and Innovation Center, DENSO CORPORATION, Nisshin, Aichi 470-0111 Japan; 5 Institute for Solid State Physics (ISSP), The University of Tokyo, Kashiwa, Chiba, 277-8581 Japan; 6 Department of Precision Engineering, Graduate School of Engineering, Osaka University, 2-1 Yamada-oka, Suita, Osaka 565-0871 Japan; 7 International Center for Materials Nanoarchitectonics, National Institute for Materials Science, Tsukuba, 305-0044 Japan

**Keywords:** Materials chemistry, Two-dimensional materials, Two-dimensional materials, Heterogeneous catalysis

## Abstract

Hydrogen boride (HB) sheets are metal-free two-dimensional materials comprising boron and hydrogen in a 1:1 stoichiometric ratio. In spite of the several advancements, the fundamental interactions between HB sheets and discrete molecules remain unclear. Here, we report the adsorption of CO_2_ and its conversion to CH_4_ and C_2_H_6_ using hydrogen-deficient HB sheets. Although fresh HB sheets did not adsorb CO_2_, hydrogen-deficient HB sheets reproducibly physisorbed CO_2_ at 297 K. The adsorption followed the Langmuir model with a saturation coverage of 2.4 × 10^−4^ mol g^−1^ and a heat of adsorption of approximately 20 kJ mol^−1^, which was supported by density functional theory calculations. When heated in a CO_2_ atmosphere, hydrogen-deficient HB began reacting with CO_2_ at 423 K. The detection of CH_4_ and C_2_H_6_ as CO_2_ reaction products in a moist atmosphere indicated that hydrogen-deficient HB promotes C–C coupling and CO_2_ conversion reactions. Our findings highlight the application potential of HB sheets as catalysts for CO_2_ conversion.

## Abstract (web summary)

Boron-containing materials have experimentally and theoretically been shown to be promising materials for CO_2_ capture and conversion. Here, hydrogen-deficient 2D hydrogen boride sheets are shown to physisorb CO_2_ and promote conversion to methane and ethane.

## Introduction

Two-dimensional (2D) materials have been applied in various fields, including catalysis and electronics, because of their large surface areas and advantageous electronic states^1–6^. We previously reported hydrogen boride (HB) sheets comprising boron and hydrogen in a 1:1 stoichiometric ratio as a 2D metal-free material that can be formed via ion-exchange reactions between the protons and magnesium cations in magnesium diboride following an exfoliation process^7^. Boron atoms form a hexagonal 2D network in the HB sheets, wherein hydrogen atoms are bound to boron atoms by 3-center-2-electron (B–H–B) and 2-center-2-electron (B–H) bonds^8^. HB sheets have been experimentally verified to exhibit excellent solid acid catalytic activity^9,10^, specific metal ion reducibility^11,12^, semimetal electronic properties^13^, highly-sensitive gas-sensor applicability^8^, and light-responsive hydrogen release^14^. Furthermore, theoretical studies have revealed the intriguing electronic^15^, optical, and thermal properties^16,17^ of HB sheets, as well as their possible applications in rechargeable Li/Na ion battery electrodes^18,19^, hydrogen release devices^20,21^, reversible hydrogen storage^22^, current limiters^23^, photodetectors^23^, individual amino acid sensors^24^, and anodes for rechargeable potassium-ion batteries with high capacities, low voltages, and high rate-performance^25^. Furthermore, the formation of HB sheets paves the way for the conceptual development of new types of HB materials^26–32^. In spite of the aforementioned advancements, the fundamental interactions between HB sheets and discrete molecules remain unclear.

In this work, we focus on the interaction between CO_2_ and HB sheets because boron is a key element associated with the activation of CO_2_ in organic chemistry^33,34^. Elemental boron has also been reported to facilitate the reduction of CO_2_ under light irradiation^35^. Theoretical calculations have indicated that CO_2_ capture on solid boron clusters is kinetically and thermodynamically feasible^36^, and that planar-type boron clusters can capture CO_2_^37^ and separate it from N_2_ and CH_4_^38^. Theoretical predictions have also indicated that 2D-planar boron (borophene) shows promise as a material for switchable charge-modulated CO_2_ capture^39,40^ and as an electrocatalyst for the conversion of CO_2_ to CH_4_^41^. B_40_ fullerene has been predicted to be an efficient material for CO_2_ capture, storage, and separation based on theoretical calculations^42,43^. Theoretical studies have also highlighted the potential of B_80_ fullerene as a metal-free photocatalyst for the efficient conversion of CO_2_ to HCOOH^44^, as well as CO_2_ capture and separation applications^45^. Borohydrides are widely used as reducing agents in chemical synthetic processes, as hydrogen storage materials in emerging energy applications, and as reagents for the reduction of CO_2_^46–50^. Boron nitride, boron sulfide, and boron phosphide have also been reported to be efficient metal-free catalysts for converting CO_2_ into valuable fuels^51–55^.

In this study, we experimentally and theoretically investigated the adsorption characteristics of CO_2_ molecules on HB sheets, and verified their utilization prospects as CO_2_ conversion catalysts. To investigate the effect of pre-treatment temperature, the HB sheets were pre-treated in vacuum for 1 h at four different temperatures (323, 473, 523, and 573 K), with the resultant samples denoted as HB-323K, HB-473K, HB-523K, and HB-573K, respectively. Further, we experimentally confirmed the formation of CH_4_ and C_2_H_6_ through the thermal reaction between hydrogen-deficient HB sheets and CO_2_ in a moist atmosphere, which indicates that hydrogen-deficient HB promotes C–C coupling in addition to CO_2_ conversion.

## Results and discussion

### CO_2_ adsorption on HB sheets

**Fig. 1:**
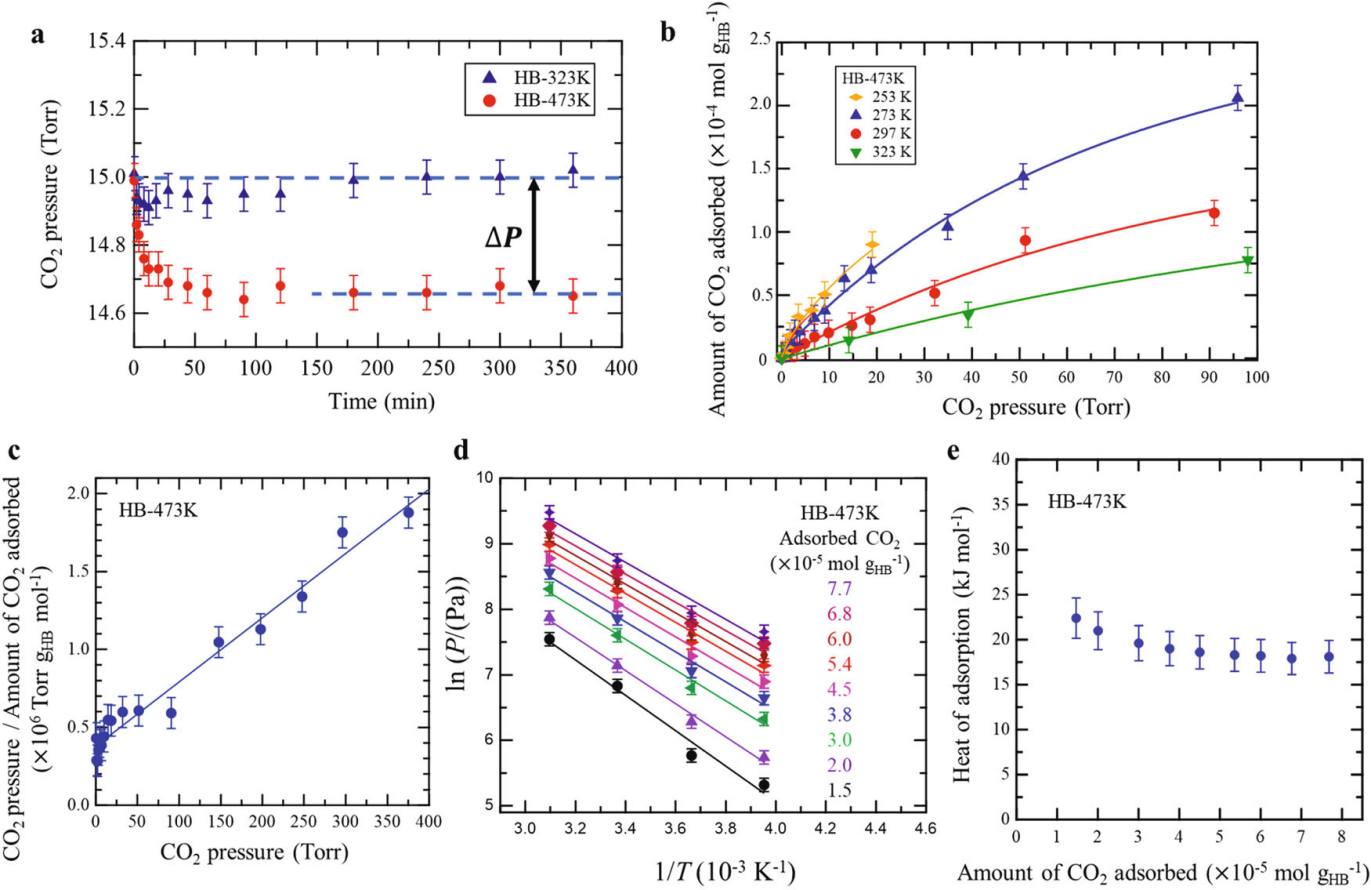
CO_2_ adsorption on hydrogen boride (HB) sheets. **a** Change in pressure of CO_2_ with time following exposure to HB sheets at 297 K. Blue triangles: HB sheets preheated at 323 K for 1 h in vacuum (HB-323K). Red circles: HB sheets preheated at 473 K for 1 h in vacuum (HB-473K), at which temperature the HB sheets became hydrogen-deficient (H_*x*_B_1_, 0.81 < *x* < 0.95) due to partial decomposition and hydrogen release (see the text for details). **b** Isotherms of CO_2_ adsorption on HB-473K. **c** *P*/*n*_ads_ vs *P* plot for the results in **b** at 273 K and the results obtained at higher CO_2_ pressures (*P*) of up to 400 Torr (*n*_ads_ denotes the amount of adsorbed CO_2_). **d** ln(*P*) vs 1/*T* plot for the results in **b**. **e** Heat of adsorption as a function of the amount of adsorbed CO_2_ derived from the Clausius–Clapeyron equation and slopes in **b**. Error bars result from the uncertainty in our measurement of pressure.

The rate of adsorption of CO_2_ on the HB sheets was determined by measuring the change in pressure following the introduction of CO_2_ into a vacuum chamber (base pressure lower than 10^−6^ Torr) containing HB sheets (30–50 mg). Typical results at 297 K and a CO_2_ pressure of 15 Torr are shown in Fig. 1a. For the HB-323K sample (blue triangles), the CO_2_ pressure remained unchanged with time, indicating that CO_2_ does not adsorb onto HB sheets pre-treated at 323 K. However, for the HB-473K sample (red circles), the CO_2_ pressure decreased with time until reaching a constant value, indicating that CO_2_ was successfully adsorbed on the HB-473K sample and attained adsorption/desorption equilibrium at 297 K. The HB-473K sample became hydrogen-deficient compared to pristine HB because a sub-stoichiometric fraction of hydrogen was released as H_2_ gas via thermal decomposition. The results in Fig. 1a illustrate the adsorption of CO_2_ when the HB sheets became hydrogen-deficient. Based on gas chromatography analysis and our previous results employing thermal desorption spectroscopy^7,9^, the fraction of hydrogen remaining in the HB-473K sample (*x* in H_*x*_B_1_) was estimated to be 0.81 < *x* < 0.95. The variation of *x* originates from the differences in the hydrogen content between samples^7^ as well as errors in temperature measurement.

The amount of adsorbed CO_2_, *n*_ads_, was estimated from the pressure drop (Δ*P*) in Fig. 1a using the ideal gas law:

$${n}_{{{{{{\rm{ads}}}}}}}=\frac{{\Delta}{PV}}{{RT}},$$

where *V* is the volume of the experimental cell, *R* is the ideal gas constant, and *T* is the temperature (297 K). We used the ideal gas law instead of the van der Waals equation because the effects of the intermolecular interactions and the volume occupied by the gas molecules were small and within the uncertainty of our experiment. The amount of CO_2_ adsorbed on HB-473K was plotted as a function of CO_2_ pressure at different sample temperatures (253–323 K) to construct the adsorption isotherms shown in Fig. 1b (also see Supplementary Fig. 1). In each case, the amount of CO_2_ adsorbed increased with increasing CO_2_ pressure and decreasing temperature. This behavior is consistent with type-I Langmuir adsorption according to the International Union of Pure and Applied Chemistry (IUPAC) classification. This indicates that the adsorption site and adsorption energy are independent of the amount of CO_2_ adsorbed.

To characterize the type of adsorption, a *P*/*n*_ads_ vs *P* plot was constructed (Fig. 1c) using the results obtained in Fig. 1b at CO_2_ pressures (*P*) of up to 400 Torr at 273 K. The linear relationship between *P*/*n*_ads_ and *P* clearly indicated that the adsorption of CO_2_ on the HB-473K sample followed a type-I Langmuir isotherm curve. The slight deviation in the low-pressure region was attributed to the small number of CO_2_ chemisorption sites on HB-473K. Specifically, 1.44 × 10^−5^ mol g_HB_^−1^ of CO_2_ was considered to be chemisorbed (Supplementary Fig. 2), whereas the majority of the CO_2_, as modeled by the type-I isotherm, remained in the physisorbed state (as discussed below). From the slope in Fig. 1c, the amount of CO_2_ adsorbed at saturation (*n*_s_) and the equilibrium constant (*K*) were estimated as *n*_s_ = 2.43 × 10^−4^ mol g_HB_^−1^ and *K* = 0.011 using the following relationship:

$$\frac{P}{V}=\frac{P}{{n}_{{{{{{\rm{s}}}}}}}}+\frac{1}{K{n}_{{{{{{\rm{s}}}}}}}}$$

The estimated *n*_s_ corresponded to 7.8 ± 6.3% of the H-vacant sites in HB-473K (H_*x*_B_1_, 0.81 < *x* < 0.95). In this calculation, we neglected the influence of the morphological characteristics of the HB sheet including sheet stacking, and assumed that all atoms in the sheets were exposed to the gas. Therefore, the estimated percentage was within a lower limit. The estimated coverage suggests that only some of the hydrogen-deficient sites in HB-473K act as adsorption sites for CO_2_.

To estimate the heat of adsorption (∆*H*) of CO_2_, the slopes of the plots of ln *P* vs 1/*T* (Fig. 1d) were analyzed using the Clausius–Clapeyron equation:

$$\Delta H=-R\frac{\partial \left({{{{{\rm{ln}}}}}}P\right)}{\partial \left({T}^{-1}\right)}$$

The results are plotted in Fig. 1e as a function of the amount of adsorbed CO_2_. The ∆*H* values (17.9–22.4 ± 2.5 kJ mol^−1^) were in the range of physisorption instead of chemisorption^56,57^.

**Fig. 2:**
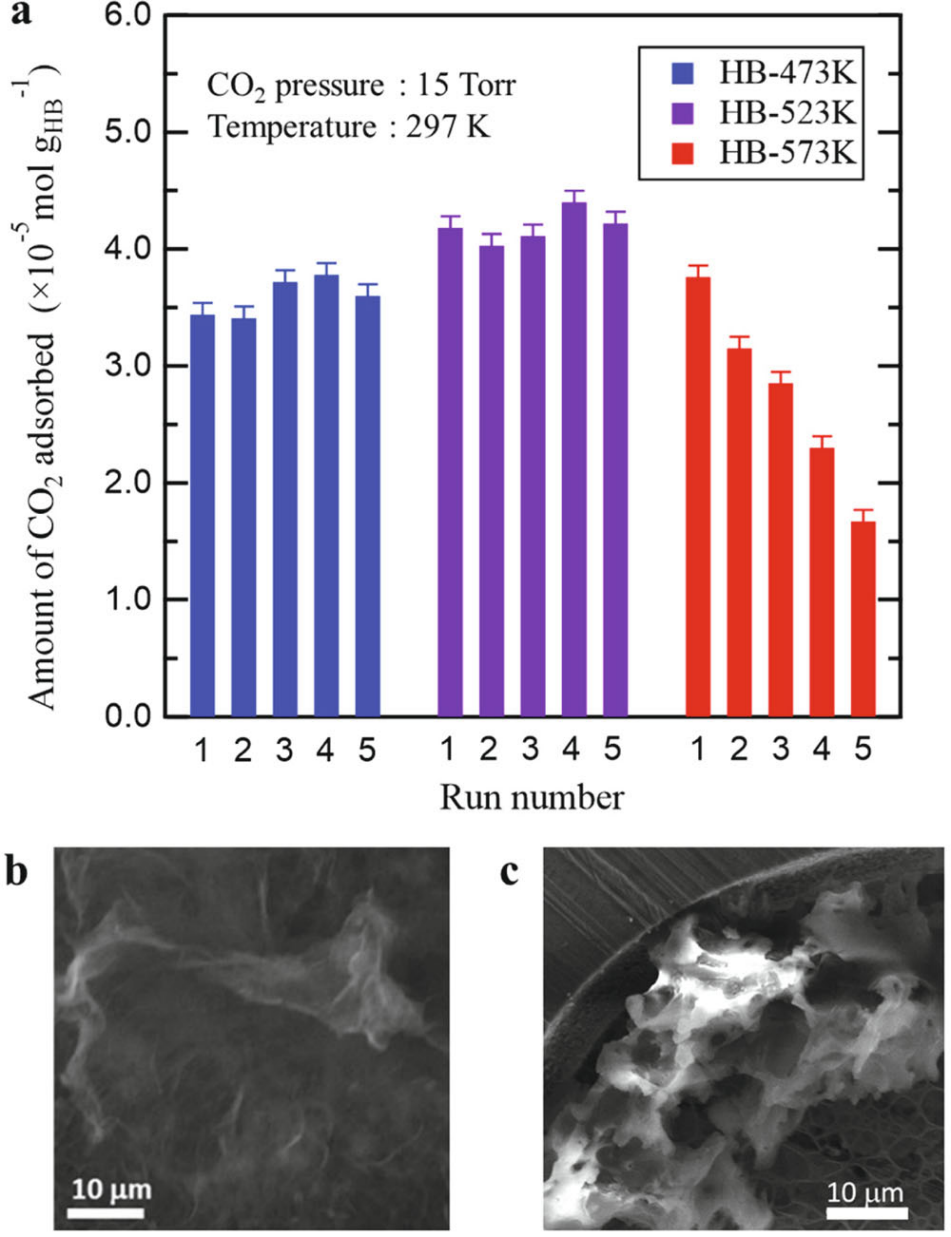
Reproducibility of CO_2_ adsorption on hydrogen boride (HB) sheets. **a** Amount of adsorbed CO_2_ plotted as a function of the number of adsorption/desorption cycles. Adsorption was performed at 297 K in CO_2_ atmosphere (15 Torr); desorption was performed for 1 h at each temperature in vacuum (473 K, 523 K, and 573 K for HB-473K, HB-523K, and HB-573K, respectively). Error bars result from the uncertainty in our measurement of pressure. **b** Scanning electron microscopy (SEM) image of pristine HB sheets. **c** SEM image of HB sheets after heating to 573 K in ultrahigh vacuum for 1 h.

Because CO_2_ is adsorbed at hydrogen vacancies, the amount of CO_2_ adsorbed was expected to increase with the number of hydrogen-deficient sites in the HB sheets (Fig. 1a). To determine the relationship between the amount of adsorbed CO_2_ and the number of hydrogen vacancy sites, and to verify the reproducibility of CO_2_ adsorption, we conducted five cycles of CO_2_ adsorption/desorption using HB-473K, HB-523K, and HB-573K samples. According to the results of our gas chromatography analysis and the findings of previously reported thermal desorption spectroscopy analyses^7,9^, the residual hydrogen occupancies (*x* in H_*x*_B_1_) for the HB-473K, HB-523K, and HB-573K samples were in the ranges of 0.81 < *x* < 0.95, 0.67 < *x* < 0.77, and 0.50 < *x* < 0.67, respectively. As described above, *x* is expressed as a range because its value is influenced by the differences in the hydrogen content between samples^7^ and the errors in temperature measurement. Figure 2a shows that the amount of adsorbed CO_2_ remained unaltered when the experiments conducted using HB-473K and HB-523K were repeated. The amount of CO_2_ adsorbed on HB-523K was greater than that on HB-473K, probably because the HB-523K sample had a greater number of hydrogen vacancy sites. However, the amount of CO_2_ adsorbed on HB-573K was lower than that on HB-473K and HB-523K. Additionally, the amount of CO_2_ adsorbed on HB-573K decreased with continued cycling, indicating that the extensively hydrogen-deficient HB sheets (H_*x*_B_1_, 0.50 < *x* < 0.67) did not support reproducible CO_2_ adsorption at 297 K.

To clarify the origin of the steady drop in CO_2_ adsorption on HB-573K during cycling, the morphology and chemical state of the HB sheets were examined by scanning electron microscopy (SEM) and X-ray photoelectron spectroscopy (XPS) before and after heating at 573 K for 1 h. The SEM images before and after heating at 573 K (Fig. 2b, c, respectively) demonstrate slight agglomeration of the HB sheets by heating, suggestive of a reduction in the number of accessible adsorption sites consequently resulting in decreased CO_2_ adsorption on HB-573K. However, the agglomeration cannot explain the steady decrease in CO_2_ adsorption amount during the cycling experiments, because the same heating condition (573 K for 1 h) was used during each cycle, and the observed morphological change is likely to have been completed after the first cycle.

Two fundamental pathways for interaction between CO_2_ and HB-573K during cycling (namely, desorption, and reaction) are suggested based on the XPS analysis (Supplementary Fig. 3). Specifically, heating at 573 K creates reactive sites in HB, some of which may be degraded by reacting with the adsorbed CO_2_, while other reactive sites survive if the adsorbed CO_2_ desorbs without reacting with them. Thus, these pathways can explain the accumulative degradation of HB-573K during cycling. Considering our previous results on the H_2_ temperature programmed desorption (TPD) of HB sheets^7^, the temperature of 573 K corresponds to the terminus of the first desorption peak and the onset of the second desorption peak. The reactive sites are thus considered to correspond to the desorption component of the second desorption peak in TPD. Therefore, although reproducible adsorption/desorption was observed for HB-473K and HB-523K, accumulative degradation of the CO_2_ adsorptivity of HB-573 K occurred during cycling (Fig. 2a).

CO_2_ molecules adsorb on HB-473K by Langmuir-type physisorption. Consequently, CO_2_ may not adsorb on the hydrogen atoms at the outermost surface (bridge- and/or edge-type hydrogen atoms) of the HB sheets; however, it may adsorb on the hydrogen vacancy sites (i.e., on boron atoms) in HB-473K. Boron atoms that are not bound to hydrogen atoms can act as Lewis acid sites, with each boron atom adopting a simple *sp*^2^ configuration comprising a vacant *p*_z_ orbital in its connection to the surrounding boron atoms. In this case, CO_2_ may physisorb in an end-on configuration with one of the oxygen atoms of CO_2_ close to the Lewis acid site of the boron atom. In contrast, if the charges in the HB sheets are sufficiently delocalized to supply electrons to the *p*_z_ orbital of a bare *sp*^2^-bonded boron atom (hydrogen vacancy), CO_2_ may physisorb in a side-on configuration with the carbon atom of CO_2_ close to the Lewis-base-like boron atom.

**Fig. 3:**
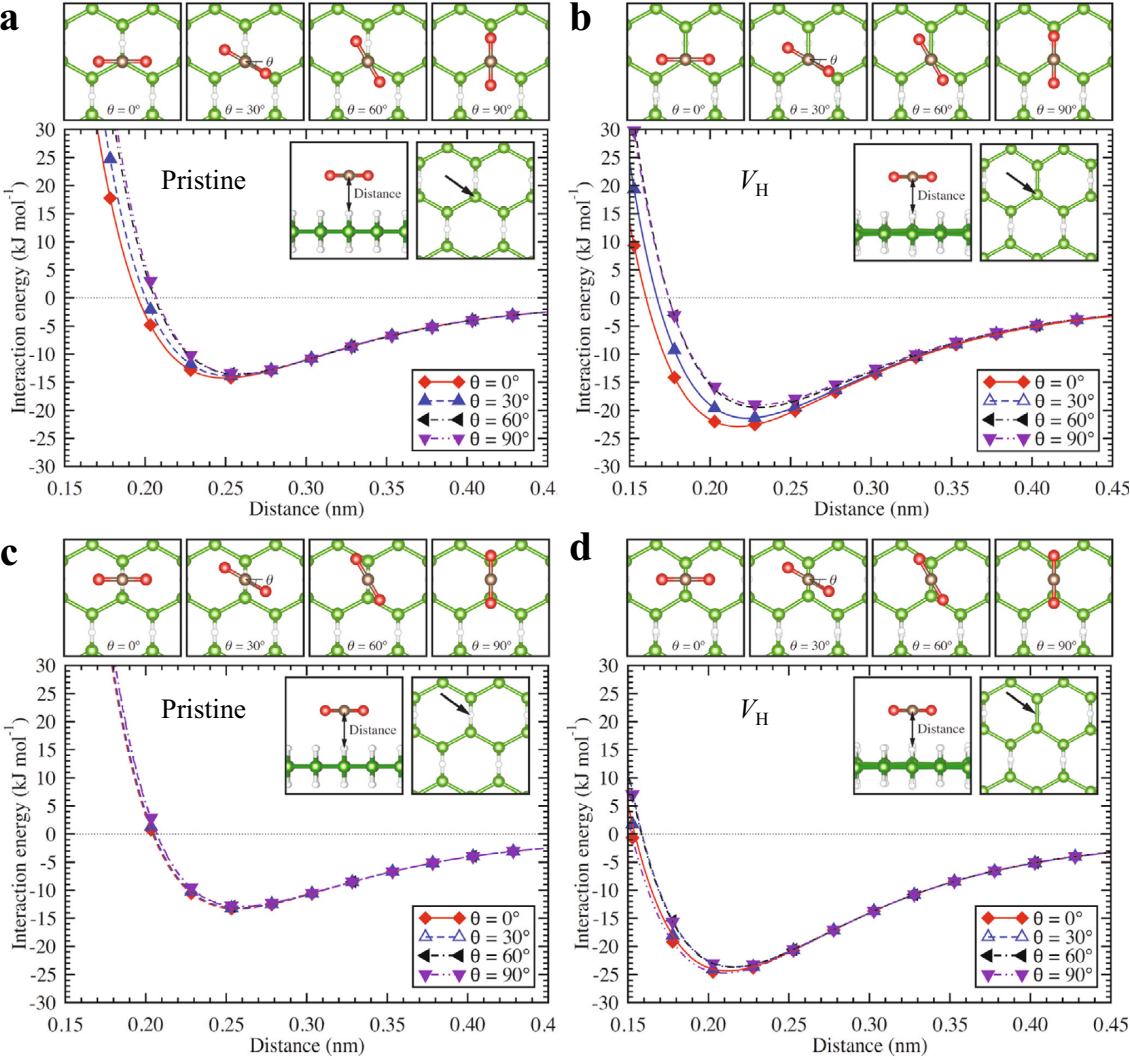
Adsorption configurations and corresponding interaction energy (*E*_int_) curves for CO_2_ adsorption on hydrogen boride (HB) sheets with the side-on configuration. **a** *E*_int_ for the on-top B atom sites of the pristine HB sheet. **b** *E*_int_ for the on-top B atom sites of the H-vacant (*V*_H_) HB sheet. **c** *E*_int_ for the on-top H sites of the pristine HB sheet. **d** *E*_int_ for the H-vacant sites of the H-vacant HB sheet. The distances are defined by the difference between the *z*-coordinate of CO_2_ and the average *z*-coordinate of the surface H atoms of the HB sheets, as indicated in the inset figures. The adsorption sites are indicated by the arrows in the insets.

We conducted density functional theory (DFT) calculations to investigate the adsorption state of CO_2_ on the HB sheets (Supplementary Figs. 4 and 5). Specifically, we calculated the potential energy surface (PES) on a grid over the primitive surface unit cell of the HB sheet by varying the distance between CO_2_ and the HB sheet and the orientation of the CO_2_ molecule with its molecular axis parallel to the surface (side-on configuration). The HB sheet and CO_2_ molecule were relaxed in their isolated states; therefore, no further relaxation was performed in the PES calculations. Thereafter, we calculated the interaction energy curves between CO_2_ and HB to determine the most stable adsorption site (Fig. 3). For the pristine HB sheet, the CO_2_ molecules were weakly adsorbed on the HB surface with an adsorption energy of 13–14 kJ mol^−1^ and no distinct preferential adsorption site or orientation with respect to the surface (Fig. 3a, c and Supplementary Fig. 4). We also calculated the PES of CO_2_ with its molecular axis perpendicular to the surface (end-on configuration) and discovered that it was less stable (Supplementary Fig. 6). These results indicated that CO_2_ weakly physisorbs onto the pristine HB sheets, and CO_2_ molecule(s) may behave like a 2D gas on the pristine HB sheet (i.e., free to rotate parallel to the surface and diffuse on HB sheets). However, when a hydrogen vacancy (*V*_H_) was introduced, distinct differences in the adsorption energy (19–25 kJ mol^−1^) were found depending on the adsorption site and molecular orientation with respect to the surface (Fig. 3b, d, and Supplementary Fig. 5). The CO_2_ molecule preferentially adsorbed at the *V*_H_ sites in the side-on configuration, with a smaller adsorption height. The calculated adsorption energy was consistent with the experimentally obtained range of 17.9–22.4 ± 2.5 kJ mol^−1^. The end-on configuration of CO_2_ was also considered and discovered to be significantly less stable than the side-on configuration with *V*_H_ (Supplementary Fig. 6).

Despite the different crystallinity states observed for the HB sheets under experimental (amorphous) and theoretical (crystalline) conditions, the calculated and experimental adsorption energies were consistent, which indicates that the CO_2_ molecules physisorb at the hydrogen vacancy sites in a side-on configuration, as shown in Fig. 3d. This implies that the boron atoms at the hydrogen-vacant sites act as weak Lewis bases in terms of charge. In particular, the charge of the HB sheets could be sufficiently delocalized to supply electrons to the *p*_z_ orbital of the bare *sp*^2^-bonded boron atom at the hydrogen vacancy site, which is consistent with the semimetal band structure of the HB sheets^7,13^.

### Thermal reaction products of HB sheets with CO_2_

Hydrocarbon molecules such as methane (CH_4_), ethane (C_2_H_6_), and propane (C_3_H_8_) are produced when HB sheets are heated in a CO_2_ atmosphere at 523 K under moist conditions (Supplementary Fig. 7 and Supplementary Table 1). Therefore, CO_2_ can be converted into hydrocarbons by reacting with the HB sheets. In this study, we focus primarily on CH_4_ and C_2_H_6_ because C_3_H_8_ was only detected in small quantities.

**Fig. 4:**
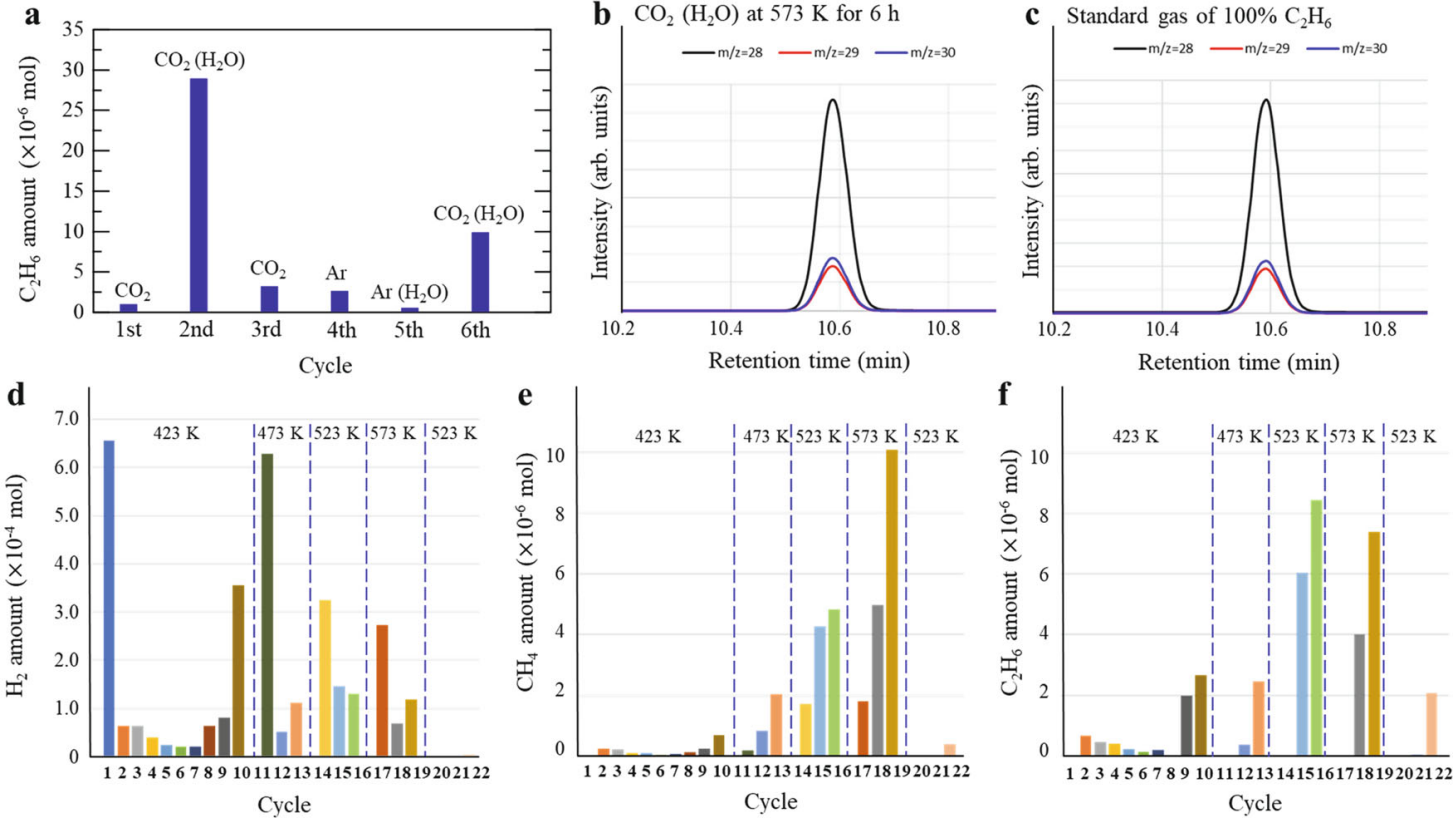
Reaction product of CO_2_ with hydrogen boride (HB) upon cycling. **a** Amount of C_2_H_6_ produced upon cycling. The labels identify the reactants supplied to the system in each cycle. Before the measurements, the HB sample was heated at 523 K for 6 h in Ar to create hydrogen vacancies in the HB sheets (100 mg). For each cycle, the gas was changed to CO_2_ or Ar at 300 K, and the HB sheets were heated at 373 K in a flow of CO_2_ or Ar. In the second, fifth, and sixth cycles, when the temperature reached 373 K, 0.1 cm^3^ of H_2_O was added to the system using a syringe. Thereafter, the reactor was closed, the gas flow was stopped, and the temperature was increased to 523 K. The sample was held at 523 K for 6 h. **b**, **c** Mass patterns of detected gas and standard gas. The reaction products were analyzed using gas chromatography-mass spectrometry (GC-MS). C_2_H_6_ was verified by comparing its GC-MS signal (**b**) with that of standard gas of 100% C_2_H_6_ (**c**). **d**–**f** Amounts of (**d**) H_2_, (**e**) CH_4_, and (**f**) C_2_H_6_ produced upon cycling by continuously using the same HB sample (100 mg). The atmospheres used for each cycle (6 h) were as follows. At 423 K: cycles 1–7, Ar gas; cycle 8, CO_2_ gas; cycles 9 and 10, CO_2_ + 0.1 cm^3^ H_2_O. At 473, 523, 573, and again 523 K: cycles 11, 14, 17, and 20, Ar flow; cycles 12, 15, 18, and 21: Ar gas; cycles 13, 16, 19, and 22, CO_2_ + 0.1 cm^3^ H_2_O. The experiments were performed from left to right along the horizontal axis.

Figure 4a shows the amount of C_2_H_6_ detected as a function of the cycle number without changing the HB sample (100 mg). The labels indicate the reactants supplied to the system in each cycle. C_2_H_6_ was verified by comparing its gas chromatography-mass spectrometry (GC-MS) signals with that of a standard gas of 100% C_2_H_6_ (Fig. 4b, c). Significant quantities of C_2_H_6_ were detected when the HB sheets were heated in CO_2_ (10 cm^3^) in the presence of H_2_O (0.1 cm^3^) at 523 K for 6 h (second and sixth cycles). Heating in CO_2_ (first and third cycles), Ar (fourth cycle), and Ar containing H_2_O (fifth cycle) produced smaller amounts of C_2_H_6_. Small quantities of C_2_H_6_ were detected in the fourth and fifth cycles although no carbon source was supplied. This observation was attributed to the residual carbon in the system from the first to the third cycles. The results described here clearly establish that the hydrogenation of CO_2_ requires both HB sheets and H_2_O. The results also indicate that the reaction is not continuous or catalytic, because the amount of C_2_H_6_ produced in the sixth cycle is lower than that produced in the second cycle.

Figures 4d–f show the results of cycling experiments at different reaction temperatures (423, 473, 523, and 573 K) to elucidate the effect of temperature. Here, each cycle run was conducted sequentially using the same HB sample. As shown in Fig. 4d, H_2_ was detected in every cycle, with the exception of the last cycle at 523 K, as a steady-state reaction product. The amount of H_2_ detected decreased as the cycle temperature increased, indicating that the hydrogen in HB is consumed by the reaction. At 423 K, CH_4_ and C_2_H_6_ were detected during cycling. The amounts of CH_4_ and C_2_H_6_ detected were largest when CO_2_ was introduced with H_2_O (cycles 9 and 10), similar to the experiment shown in Fig. 4a. The amount of CH_4_ detected increased with increasing reaction temperature, while that of C_2_H_6_ started to decrease at 573 K. These results indicate that the reaction between CO_2_ and H in the HB sheets, including C–C coupling, starts to occur even at the relatively low temperature of 423 K. Furthermore, the amount of CH_4_ produced exceeds that of C_2_H_6_ at 573 K, indicating that the rate of CH_4_ desorption is faster than the rate of C–C coupling at 573 K. Finally, the fact that the final cycle at 523 K does not show reproducibility suggests that degradation reaction may be occurring due to insufficient amount of hydrogen in the HB sheets.

The aforementioned results indicate that the presence of HB promotes C–C coupling and CO_2_ conversion, considering that the products originate not only from simple hydrogenation using the hydrogen in HB, but also from bond rearrangements, such as C–C coupling. Furthermore, the detection of CO (Supplementary Fig. 7a and Supplementary Table 1) indicates that the dissociation of CO_2_ can also occur on the HB sheets. The possible reaction steps are as follows:

1 $$({{{\mathrm{H}}}}_{1}{{{\mathrm{B}}}}_{1})_{n}\to ({{{\rm{H}}}}_{{{\mathrm{x}}}}{{{\mathrm{B}}}}_{1})_{n}+({n/2}){{{\mathrm{H}}}}_{2}$$

2 $${{{\mathrm{CO}}}}_{2}({{{\mathrm{g}}}})\to {{{\mathrm{CO}}}}_{2}({{{\mathrm{a}}}})$$

3a $${{{\mathrm{CO}}}}_{2}({{{\mathrm{a}}}})\to {{{\mathrm{CO}}}}({{{\mathrm{g}}}})+{{{\mathrm{O}}}}({{{\mathrm{a}}}})$$

3b $${{{\mathrm{CO}}}}_{2}({{{\mathrm{a}}}})\to {{{\mathrm{CO}}}}({{{\mathrm{a}}}})+{{{\mathrm{O}}}}({{{\mathrm{a}}}})$$

4 $${{{\mathrm{CO}}}}({{{\mathrm{a}}}})\to {{{\mathrm{C}}}}({{{\mathrm{a}}}})+{{{\mathrm{O}}}}({{{\mathrm{a}}}})$$

5 $${{{\mathrm{C}}}}({{{\mathrm{a}}}})+{{{\mathrm{3H}}}}({{{\mathrm{in}}}}({{{\mathrm{H}}}}_{{{\mathrm{x}}}}{{{\mathrm{B}}}}_{1})_{{{\mathrm{n}}}})\to {{{\mathrm{CH}}}}_{3}({{{\mathrm{a}}}})$$

6a $${{{\mathrm{CH}}}_{3}}({{{\mathrm{a}}}})+{{{\mathrm{H}}}}({{{\mathrm{in}}}}({{{\mathrm{H}}}}_{{{\mathrm{x}}}}{{{\mathrm{B}}}}_{1})_{{{\mathrm{n}}}})\to {{{\mathrm{CH}}}}_{4}({{{\mathrm{g}}}})$$

6b $${{{\mathrm{CH}}}}_{3}({{{\mathrm{a}}}})+{{{\mathrm{CH}}}}_{3}({{{\mathrm{a}}}})\to {{{\mathrm{C}}}}_{2}{{{\mathrm{H}}}}_{6}({{{\mathrm{g}}}})$$

where (H_1_B_1_)_*n*_ and (H_*x*_B_1_)_*n*_ denote pristine and hydrogen deficient HB sheets, respectively, (*g*) and (*a*) represent the gas and adsorbed states, respectively, and 0.67 < *x* < 0.77. Reactions (1) and (2) are supported by the results in Fig. 1. Reaction (3a) is supported by the detection of CO (Supplementary Figs. 7a, and 8a and Supplementary Table 1). Reactions (6a) and (6b) are supported by the results in Figs. 4d–f.

**Fig. 5:**
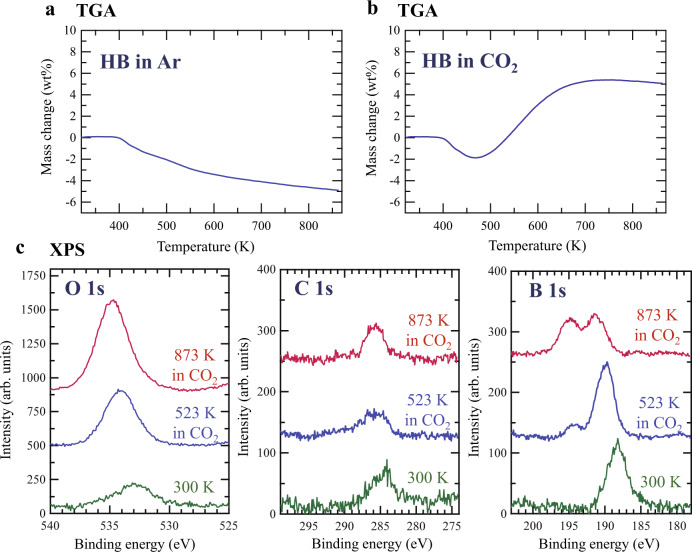
Changes in hydrogen boride (HB) sheets upon heating under various reaction conditions. Thermogravimetric analysis (TGA) of the HB sheets in flows of **a** Ar and **b** CO_2_. **c** O 1s, C 1s, and B 1s XPS spectra of HB sheets at 300 K following heating at 523 K in CO_2_, and at 873 K in CO_2_.

To investigate the presence of residual reaction products on the HB sheets, such as O(a) in reaction (3), we conducted thermogravimetric analysis (TGA) of the HB sheets under Ar or CO_2_ flow and performed post-experimental analysis of the samples by XPS and X-ray diffraction (XRD) analyses, the results of which are shown in Fig. 5 and Supplementary Figs. 9 and 10. When the HB sheets were heated in Ar, the weight of the sample began decreasing at 400 K (Fig. 5a). By comparing with our previously reported thermal desorption spectroscopy results^7,9^, this change was attributed to hydrogen release. When heated under CO_2_ flow, the weight of the sample still began decreasing at 400 K, as in the case of Ar flow; however, it subsequently began to increase at 466 K (Fig. 5b). The net increase in weight reached a maximum of 5.4% at 734 K. This increase indicates that HB reacted with CO_2_ and that the reaction products remaining on the sample compensated for the weight of lost hydrogen. The post-experimental XPS analysis of the samples indicated that oxygen was the principal element remaining on the HB sheets (Fig. 5c). The intensity of the O 1s peak clearly increased with increasing treatment temperature, whereas the intensity of the C 1s peak remained unchanged. The XPS results were consistent with the process proposed in reactions (4)–(6), wherein oxygen remained on the surface while carbon was desorbed as CH_4_ or C_2_H_6_. The C 1s and O 1s XPS peaks were detected even for fresh HB sheets at 300 K, and were attributed to the adventitious carbon and traces of oxygen on the Au substrate. Overall, the XPS survey scan and XRD results verified that the sample was free of impurities (Supplementary Figs. 9 and 10).

There are two possible roles for H_2_O in the conversion of CO_2_: (i) as a promoter of reactions (3)–(6) by forming a specific, perhaps catalytic, intermediate; and (ii) as a hydrogen source for the hydrogen vacancies in the HB sheets. Recent experiments have shown that proton exchange occurs on HB sheets in liquid water^58^. Therefore, water may accept a proton from HB to form H_3_O^+^, which can participate in redox reactions accompanied by electron transfer within HB, or in proton transfer reactions without the application of an external field, as in the rusting of iron. The identification of water as the hydrogen source in the hydrogenation reaction is supported by the effect of H_2_O on the conversion of ethanol on hydrogen-deficient HB sheets^9^. In the previous study, the presence of water influenced the product distribution by extensively promoting hydrogenation, which increased the selectivity toward C_2_H_6_ relative to C_2_H_4_^9^. We plan to examine the role of water and the proposed reaction pathways (1)–(6) using in situ spectroscopy with collective knowledge from theoretical calculations in our future work.

The detection of C_2_H_6_ as a CO_2_ reaction product demonstrates that the hydrogen-deficient HB sheets significantly promoted C–C coupling and CO_2_-conversion reactions. Both CO_2_ conversion^33,34^ and C–C coupling^59,60^ have been observed in boron-based homogeneous organic molecular catalysts, wherein the strong Lewis acidity and low electronegativity of boron permit it to mediate C–C coupling. However, our results clearly demonstrate that a solid material comprised solely of boron and hydrogen can accomplish both C–C coupling and CO_2_ conversion. It is likely that some of the hydrogen-deficient sites (boron sites) of HB sheets possess similar electronic states to the active sites in boron-based homogeneous organic molecular catalysts. In any case, the findings here indicate that HB sheets may be employed as a heterogeneous catalyst or catalyst support for CO_2_ conversion. This finding requires further analysis and system optimization, including the addition of an appropriate hydrogen source.

## Conclusion

In this work, we examined the adsorption and conversion efficiency of CO_2_ using HB sheets. Whereas fresh HB failed to adsorb CO_2_ at 297 K, hydrogen-deficient HB sheets preheated at 473 K (H_*x*_B_1_, 0.81 < *x* < 0.98) reproducibly physisorbed CO_2_. The adsorption mechanism followed the Langmuir model with saturation coverage of 2.4 × 10^−4^ mol g^−1^ at 297 K and heat of adsorption of ~20 kJ mol^−1^. This behavior suggests that only some of the hydrogen vacancy sites in the hydrogen-deficient HB sheets provide suitable sites for CO_2_ adsorption. In contrast, the extensively hydrogen-deficient HB sheet (H_*x*_B_1_, 0.50 < *x* < 0.67) did not show reproducible CO_2_ adsorption performance at 297 K, presumably due to the chemical changes caused by the release of an excessive amount of hydrogen upon heating. When a fresh HB sheet was heated in a flow of CO_2_, HB began reacting with CO_2_ at 423 K in its hydrogen-deficient state. Notably, at 423 K and under a moist atmosphere, CH_4_ and C_2_H_6_ were detected as the products of the reaction between CO_2_ and the HB sheets, indicating that hydrogen-deficient HB promotes both C–C coupling and CO_2_ conversion reactions. Although CO_2_ conversion^33,34^ and C–C coupling^59,60^ have been previously exhibited by boron-based molecular systems, our results show that a solid material comprised solely of boron and hydrogen can accomplish both C–C coupling and CO_2_ conversion. Our results also indicate that HB sheets bear significant potential as heterogeneous catalysts or catalyst supports for CO_2_ conversion. However, further detailed analysis is required for the design of optimized systems, including the selection of an appropriate hydrogen source or supply, with the objective of achieving continuous catalytic CO_2_ conversion.

## Methods

### Synthesis of HB sheets

The HB sheets were prepared using previously reported ion-exchange methods^7–11,14^. Specifically, MgB_2_ powder (1.0 g, 99%, Rare Metallic Co., Ltd., Tokyo, Japan) in acetonitrile (300 mL, 99.5%, Wako Pure Chemical Industries Ltd., Osaka, Japan) was added to a mixture of an ion-exchange resin (60 mL, Amberlite IR120B hydrogen, Organo Corp., Tokyo, Japan) and acetonitrile (200 mL) in a Schlenk flask in a nitrogen atmosphere. Water was carefully removed due to the facile hydrolysis of MgB_2_^61^. The resulting mixture was stirred with a magnetic stirrer at 400 rpm for 3 d at room temperature (~300 K). We did not apply the recently reported acid-assisted reaction^62^. The reaction mixture was allowed to settle for a sufficient time (a few hours), whereafter the supernatant was collected and stored for 1 d at 255 K to physically separate the B(OH)_3_ by-product. For samples containing unreacted materials such as the oxides present in the starting materials, the reaction mixture was filtered through a 0.2-μm pore filter (Omnipore Membrane Filters, Merck Millipore, Billerica, MA, USA), and the filtrate was stored for 1 d at 255 K to physically separate the B(OH)_3_ by-product. The dried HB sheets were prepared by heating the resulting liquid at 343 K, and the gas that evaporated during heating was pumped through a liquid nitrogen trap. We characterized the product rigorously using XPS (JPS 9010 TR, JEOL, Ltd., Tokyo, Japan) to confirm the absence of Mg, the presence of negatively charged B, and the absence of oxidized B^7–11,14^.

### CO_2_ adsorption measurements

CO_2_ adsorption measurements were conducted using a custom-made experimental system. The HB sample (30–50 mg) was placed at the bottom of a quartz tube in a He atmosphere and evacuated to a high vacuum (~10^−6^ Torr) using a Hickman-type oil diffusion pump (Makuhari Rikagaku Glass Inc., Chiba, Japan) and an oil rotary pump (GVD-165A, ULVAC, Japan) via a liquid nitrogen trap. The pressure was monitored using a capacitance manometer (Baratron Type622, MKS Instruments Japan Co., Tokyo, Japan) and a Pirani vacuum gauge (Wakaida Science Co., Tokyo, Japan). Both pressure gauges were connected to a WVG-1T unit (Wakaida Science Co., Tokyo, Japan) to display the pressure. The sample temperature was monitored using a type-K thermocouple attached to the exterior of the quartz glass close to the sample. The sample temperature was controlled using an electric heater equipped with a temperature controller (RTC5630, Okura Ltd., Japan). We used ice water to achieve a temperature of 273 K, and a mixture of ethanol, ice (400 mL), and NaCl (15 g) to achieve a temperature of 253 K.

### Analysis of thermal reaction products

The thermal reaction products between the HB sheets and CO_2_ were examined using a home-built experimental system. Fresh HB sheets (100 mg) were placed in a quartz tube (10 cm^3^) with quartz wool and heated to 523 K in a flow of CO_2_. After reaching 523 K, we closed the valves on the CO_2_ flow line to achieve a batch reaction system. This method was employed for the experiments shown in Supplementary Fig. 6 and Supplementary Table 1; whereas, for the experiments shown in Fig. 4 and Supplementary Fig. 7, the valves were closed at 373 K to avoid the flow of the products during heating to 573 K. After closing the valves, we introduced 0.1 cm^3^ of distilled water into the system using a syringe because the introduction of water enhanced the conversion of CO_2_ (see main text ‘Thermal reaction products of HB sheets with CO_2_’). After 6 h, we extracted the interior gas (1.0 cm^3^) using a syringe. The extracted gas was analyzed using a gas chromatographer (GC-8A, Shimadzu Corporation, Ltd., Kyoto, Japan) equipped with 5A molecular sieves (60–80 mesh, GL Sciences, Inc.) and a Porapak Q column (50−80 mesh, Waters Chromatography Ireland Ltd.). We also examined the gas samples via GC-MS (Shimadzu GC-MS-QP2010 Plus, Shimadzu Corporation, Ltd., Kyoto, Japan) using a GC equipped with a Shimadzu SH-Rt-Msieve 5A column. Helium was used as the carrier gas, and supplied at 50.4 cm s^−1^. When we continued to use the same HB sample, the gas inside the tube was replaced using He (for the experiments shown in Supplementary Fig. 6 and Supplementary Table 1), Ar, or CO_2_ (the gas used for the subsequent experimental cycle shown in Fig. 4 and Supplementary Fig. 7) at room temperature (~300 K), and finally replaced with CO_2_.

### X-ray photoelectron spectroscopy

XPS measurements were conducted at room temperature (~298 K) using a JPS 9010 TR spectrometer (JEOL, Ltd., Japan) equipped with an ultrahigh vacuum chamber and an Al Kα X-ray source (1486.6 eV). The pass energy was 10 eV, the energy resolution (estimated from the Ag 3d_5/2_ peak width of a clean Ag sample) was 0.635 eV, and the binding energy uncertainty was ±0.05 eV. The sample was placed on an Au surface (Au sheet, Au-173421, 99.95%, Nilaco Co. Ltd.). The Shirley background was subtracted from the spectrum using SpecSurf version 1.8.3.7 (JEOL, Ltd., Japan). The charge build-up in the sample (due to the incomplete contact of the Au sheet with the sample holder of the apparatus) caused a slight shift to higher binding energies for those spectra. Therefore, we calibrated the charge build-up based on the Au 4f_7/2_ peak as 84.0 eV.

### Thermogravimetric analysis

TGA was performed using an STA 2500 Regulus apparatus (Netzsch Japan, Ltd., Japan). Initially, ~10 mg of HB sheets were placed in an Al_2_O_3_ crucible in the apparatus under vacuum, which was followed by the introduction of Ar at 20 mL min^−1^. Thereafter, the sample was heated at 373 K for 30 min in a 10 mL min^−1^ flow of Ar to remove the adsorbed water, and subsequently cooled to 323 K. Finally, the TGA measurements were performed up to 873 K at a heating rate of 10 K min^−1^ in a 100 mL min^−1^ flow of Ar or CO_2_.

### X-ray diffraction

The XRD patterns were recorded at room temperature (~300 K) using a benchtop X-ray diffractometer (Rigaku MiniFlex, Tokyo, Japan) employing Cu Kα radiation. The X-rays were generated using the line focus principle. A reflection-free Si plate was used as the sample stage. The diffraction patterns were recorded using a D/teX Ultra silicon strip detector (Rigaku) at 0.01° s^−1^ up to a 2θ value of 90°.

### Scanning electron microscopy

SEM measurements were performed on a JSM-521 (JEOL, Ltd., Japan) operating at 10 kV. Samples were placed on Cu-TEM grids.

### Density functional theory

Periodic DFT calculations were performed using the Quantum-ESPRESSO package^63–66^. The electron–ion interactions were described using the GBRV^67^ ultrasoft pseudopotentials^68^ and the wave functions were expanded in terms of the plane-wave basis set. The rev-vdW-DF2^69,70^ functional was used for the exchange correlation. A CO_2_ molecule was placed in a 4 × 4 supercell, and the effective screening medium method^71,72^ was used to eliminate spurious electrostatic interactions between neighboring HB sheets. Further details can be found in ref. ^58^ . The interaction (potential) energy (*E*_int_) was defined as *E*_int_ = *E*_tot_(CO_2_/HB)−*E*_tot_(CO_2_)−*E*_tot_(HB), where *E*_tot_(CO_2_/HB), *E*_tot_(CO_2_), and *E*_tot_(HB) are the total energies of the adsorption system, CO_2_ molecules in the gas phase, and free-standing HB sheets, respectively. The adsorption energy was defined as the negative of the interaction energy (−*E*_int_) at the equilibrium height.

## Supplementary information

Supplemental material

## Data Availability

All results are reported in the main paper and Supplementary Information. Input and output files for Quantum-ESPRESSO^63–66^ calculations are available on the Materials Cloud Archive^73^. All other data are available from the authors upon reasonable request.
