# Supplementary material for: Carbon dioxide adsorption and conversion to methane and ethane on hydrogen boride sheets
Source: Commun Chem. 2022 Oct 4;5:118. doi: 10.1038/s42004-022-00739-8 (PMC9814476; doi:10.1038/s42004-022-00739-8)
Supplement: Supplementary file 1 — Supplemental material [file 42004_2022_739_MOESM1_ESM.pdf]

SUPPLEMENTARY INFORMATION

Carbon dioxide adsorption and conversion to methane and ethane on hydrogen boride sheets

Taiga Goto[1], Shin-ichi Ito[2,3], Satish Laxman Shinde[3], Ryota Ishibiki[1], Yasuyuki Hikita[4], Iwao Matsuda[5], Ikutaro Hamada[6]*, Hideo Hosono[2,7], and Takahiro Kondo[2,3]*

[1] Graduate School of Pure and Applied Sciences, University of Tsukuba, 1-1-1, Tennodai, Tsukuba 305-8573, Japan

[2] Materials Research Center for Element Strategy, Tokyo Institute of Technology, Yokohama 226-8503, Japan

[3] Department of Materials Science and Tsukuba Research Center for Energy Materials Science, Faculty of Pure and Applied Sciences, University of Tsukuba, Tsukuba 305-8573, Japan

[4] Advanced Research and Innovation Center, DENSO CORPORATION, Nisshin, Aichi 470-0111, Japan

[5] Institute for Solid State Physics (ISSP), The University of Tokyo, Kashiwa, Chiba 277-8581, Japan

[6] Department of Precision Engineering, Graduate School of Engineering, Osaka University, 2-1 Yamada-oka, Suita, Osaka 565-0871, Japan

[7] International Center for Materials Nanoarchitectonics, National Institute for Materials Science, Tsukuba 305-0044, Japan

*Correspondence to: ihamada@prec.eng.osaka-u.ac.jp (I. Hamada) and takahiro@ims.tsukuba.ac.jp (T. Kondo)

This file includes:

Supplementary Figure 1: CO_2 pressure changes over time at different hydrogen boride (HB) sheet temperatures.

Supplementary Figure 2: Estimation of chemisorbed CO_2.

Supplementary Figure 3: X-ray photoelectron spectra of HB sheets.

Supplementary Figure 4: Potential energy surfaces for CO_2 in the side-on configuration on a pristine HB sheet at various CO_2–HB sheet distances (z) and molecular orientations (θ).

Supplementary Figure 5: Potential energy surfaces for CO_2 in the side-on configuration on a H-vacant HB sheet at various CO_2–HB sheet distances (z) and molecular orientations (θ).

Supplementary Figure 6: Potential energy surfaces for CO_2 in the end-on configuration on pristine and H-vacant HB sheets (V_H) at various CO_2–HB sheet distances (z).

Supplementary Figure 7: Reaction products of CO_2 (10 cm^3) and HB sheets (100 mg) at 523 K under moist conditions (0.1 cm^3 H_2O).

Supplementary Table 1: Detected gas species and amounts detected 350 min after the temperature of the HB sheets in the CO_2 system reached 523 K.

Supplementary Figure 8: Reaction products of CO_2 (10 cm^3) and HB sheets (100 mg) after 6 h at 523 K under moist conditions (0.1 cm^3 H_2O).

Supplementary Figure 9: X-ray photoelectron spectroscopy survey scan of HB at 300 K, HB after heating at 523 K in CO_2, and after heating at 873 K in CO_2.

Supplementary Figure 10. X-ray diffraction (XRD) of HB at 300 K, after heating at 523 K in CO_2, and after heating at 873 K in CO_2.

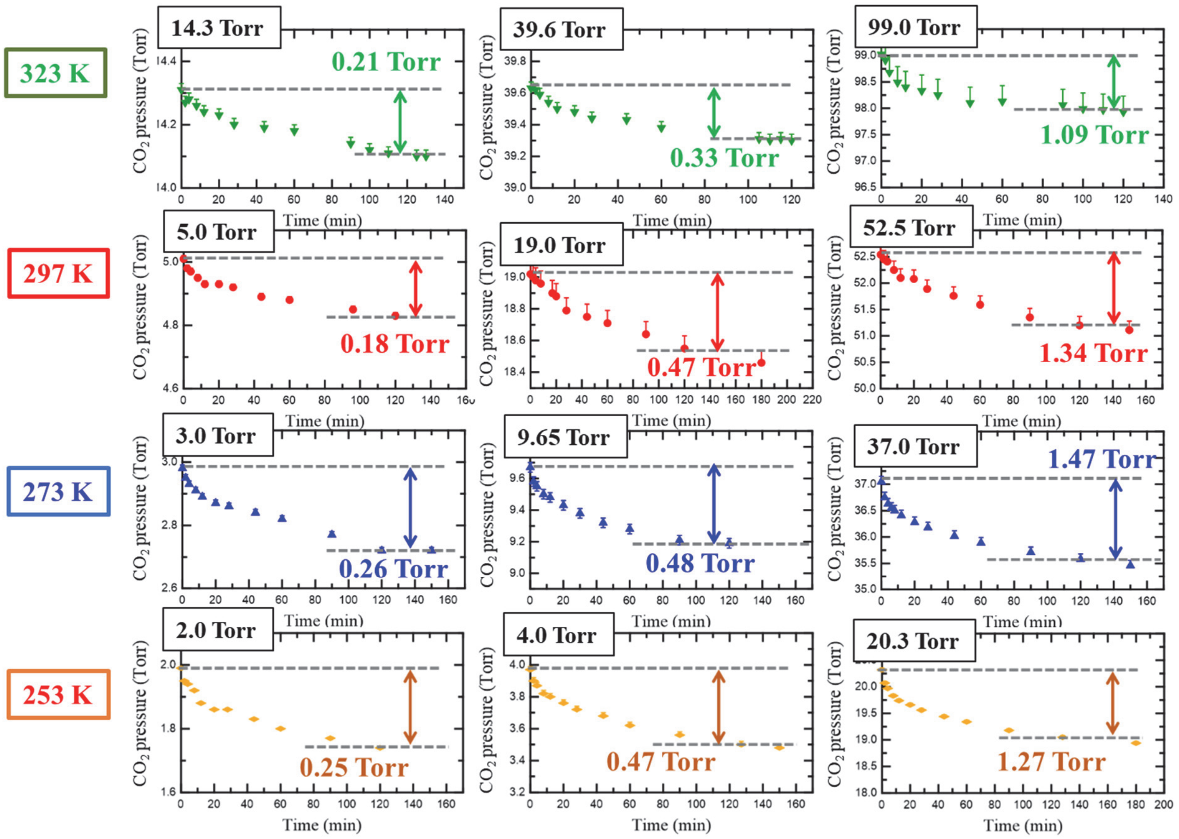

Supplementary Figure 1 | CO_2 pressure changes over time at different hydrogen boride (HB) sheet temperatures. Error bars result from the uncertainty in our measurement of pressure.

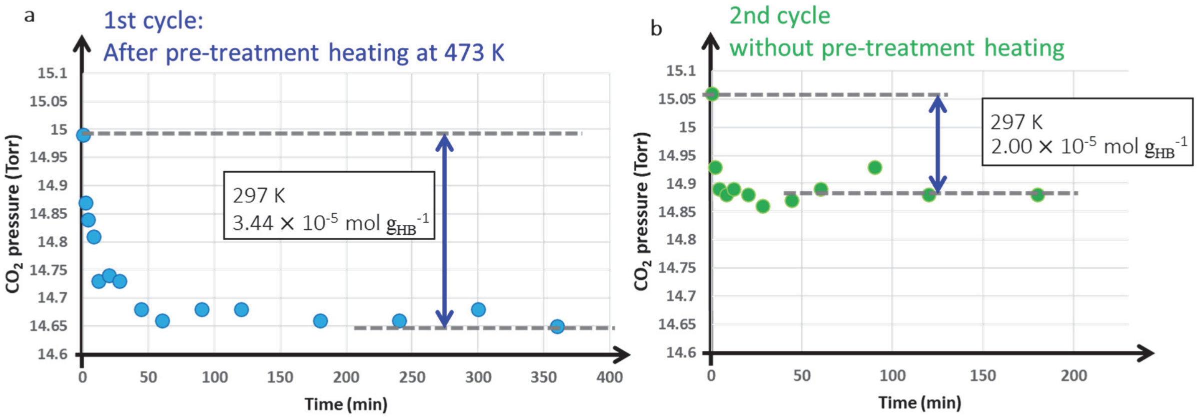

Supplementary Figure 2 | Estimation of chemisorbed CO_2. a) CO_2 pressure change at 297 K for HB sheets pre-heated at 473 K under vacuum for 1 h (first cycle). **b)** CO_2 pressure change at 297 K for HB sheets after the first cycle, where instead of conducting the pre-heating process, the gas left in the system was simply evacuated (second cycle). The pressure change in the second cycle did not resemble that in the first cycle, which contradicted the case shown in **Fig. 2**. In this case, because there was already chemisorbed CO_2 on the sample, the pressure change due to physisorption was small. The amount of chemisorbed CO_2 was thus estimated as 1.44×10^{-5} mol g_{HB}^{-1} by subtraction of the observed physisorbed CO_2 amount.

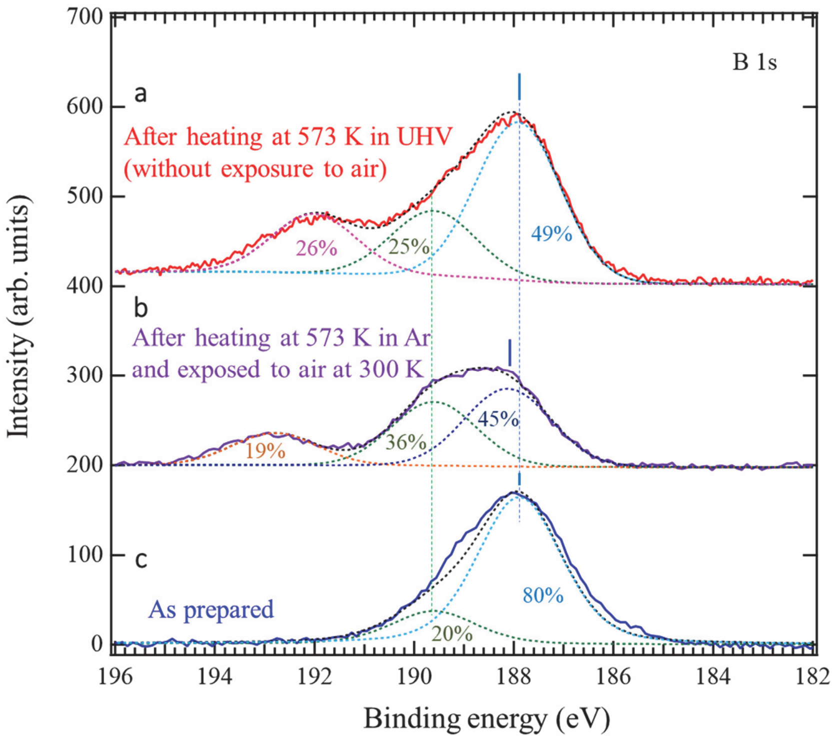

Supplementary Figure 3 | X-ray photoelectron spectra of HB sheets. B 1s spectra of (**a**) HB sheets after heating at 573 K in ultra-high vacuum (UHV), (**b**) HB sheets after heating at 573 K followed by exposure to air at 300 K and evacuated to UHV, and (**c**) as-prepared HB sheets. Compared to the as-prepared HB sheets, the main peak at 187.9 eV (corresponding to $B^{\delta-}$) decreased from 80% to 49% after heating without air exposure (peak at 188.8 eV may correspond to the B at defects and/or edges). In addition, a peak appeared at 192 eV corresponding to an oxidized component. This indicates that 31% of the boron in the HB sheets changed its chemical state upon heating at 573 K, which corresponds to H_2 release. After air exposure at 300 K and evacuation to UHV, most of the $B^{\delta-}$ peak component shifted, indicating that the chemical nature of all boron species in the sample changed upon air exposure owing to the adsorption of molecules at the reactive sites. Some of the boron species may recover their original states after the desorpiton of these adsorbed species; however, some may fail to recover, thus causing the HB sheets to degrade. These results suggest that the hydrogen-deficient HB sheets prepared by heating at 573 K are chemically unstable and react readily with residual oxygen, CO_2, and/or water in the system to form oxidized boron, which hinders the surface adsorption of CO_2.

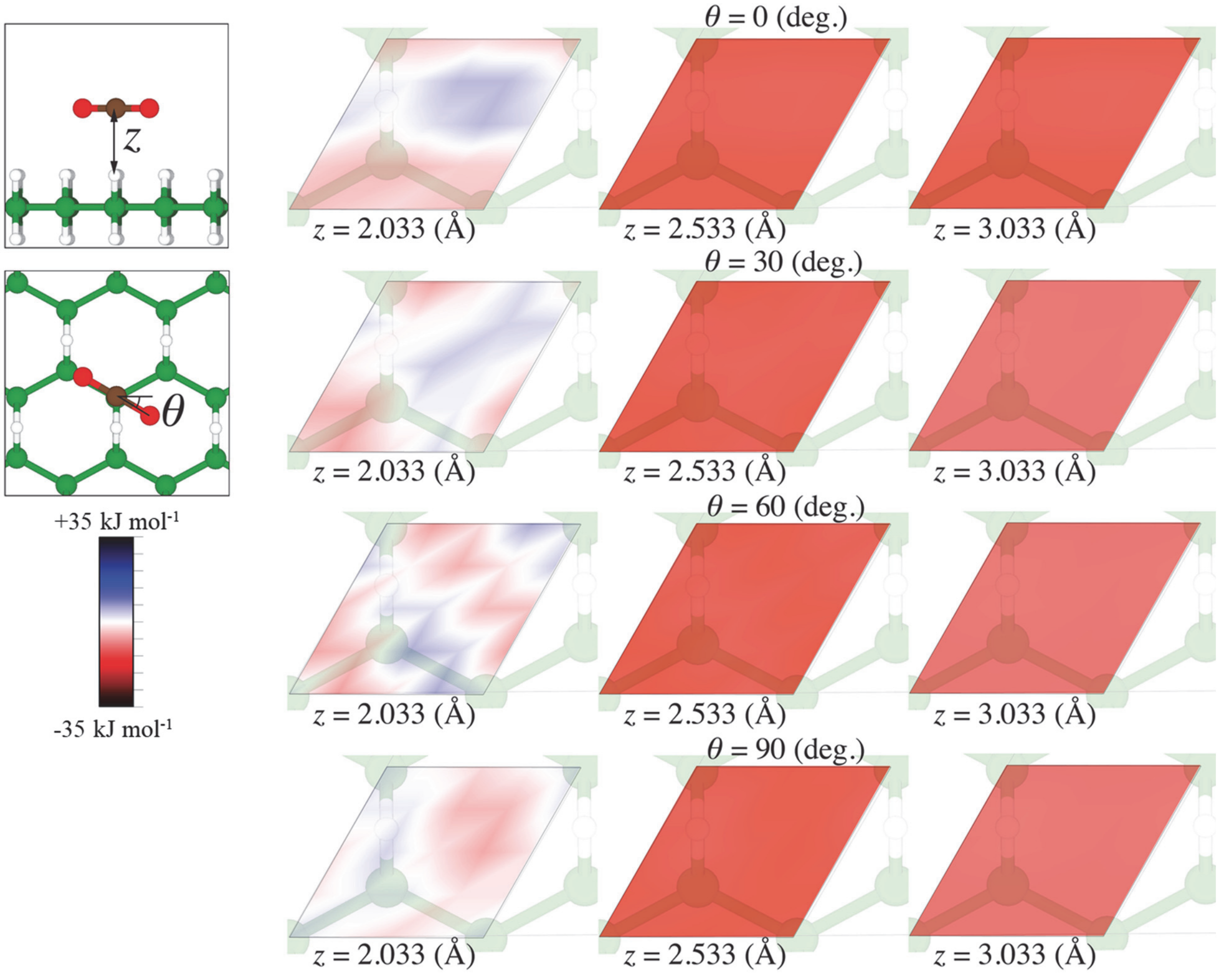

Supplementary Figure 4 | Potential energy surfaces for CO_2 in the side-on configuration on a pristine HB sheet at various CO_2–HB sheet distances (*z*) and molecular orientations (*θ*). The adsorption configuration is shown on the left: *z* is defined by the difference between the *z*-coordinate of CO_2 and the average *z*-coordinate of the surface H atoms of the HB sheet. In the calculations, the C atom of CO_2 was placed on a 4 × 4 grid of the primitive surface unit cell of the HB sheet. The values of adsorption energy are shown in the bar on the left, where blue (or red) indicates the repulsive (or attractive) interaction between CO_2 and the HB sheet. At a short distance (z = 2.033 Å), the repulsive interaction dominates as indicated by the blue iso-surfaces, whereas at larger distances, the interaction is attractive as indicated by the red iso-surface; however, there is no significant preference for the adsorption site or orientation.

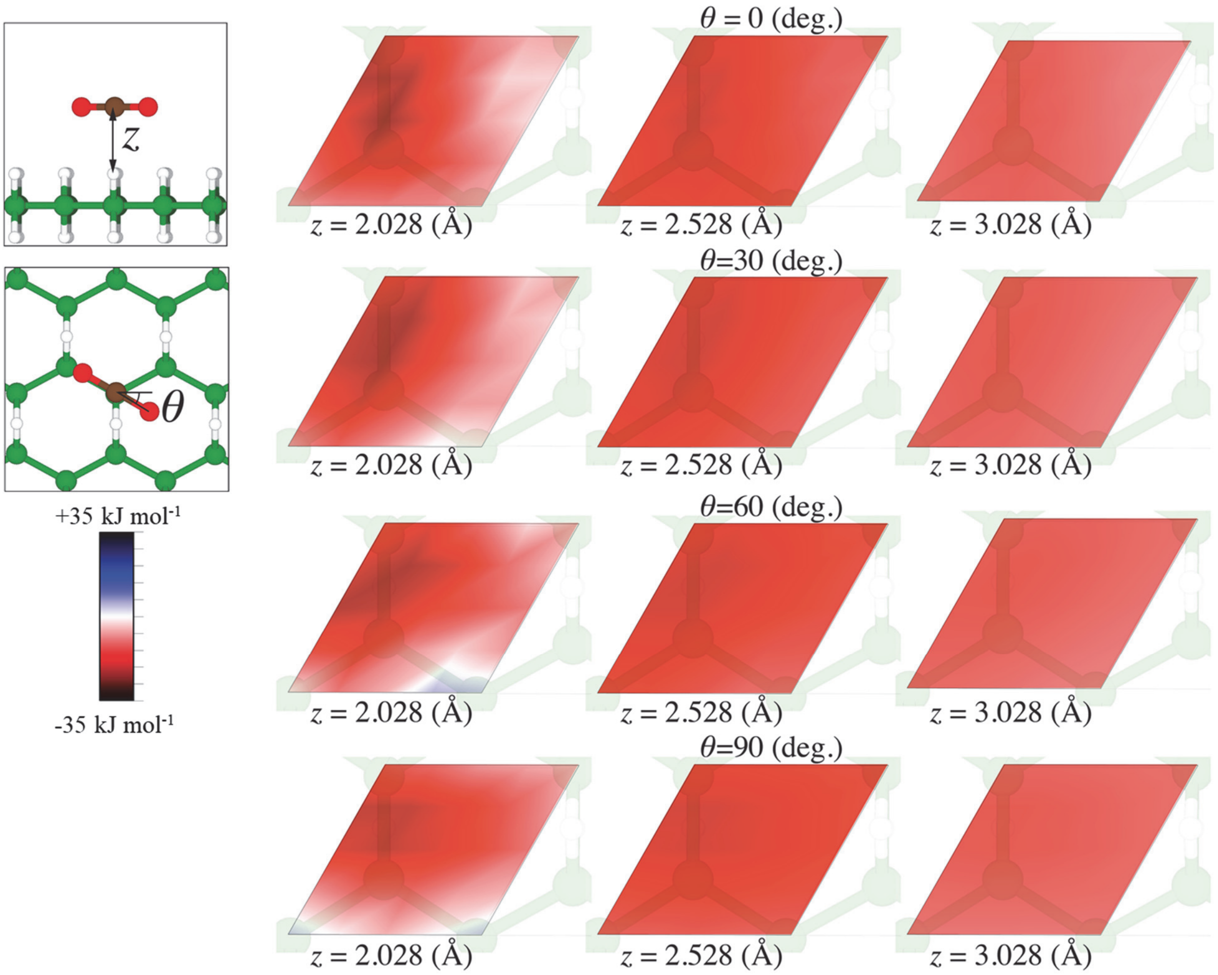

Supplementary Figure 5 | Potential energy surfaces for CO_2 adsorption in the side-on configuration on a H-vacant HB sheet at various CO_2–HB sheet distances (*z*) and molecular orientations (*θ*). The adsorption configuration is shown on the left: *z* is defined by the difference between the *z*-coordinate of CO_2 and the average *z*-coordinate of the surface H atoms on HB. In the calculation, the C atom of CO_2 was placed on a 4 × 4 grid of the primitive surface unit cell of the HB sheet. The values of the adsorption energy are shown in the bar on the left, where blue (or red) indicates the repulsive (or attractive) interaction of CO_2 with the HB sheet. At a short distance (z = 2.028 Å), there is a preferential adsorption at the H vacancy site, which is indicated by the dark red iso-surfaces.

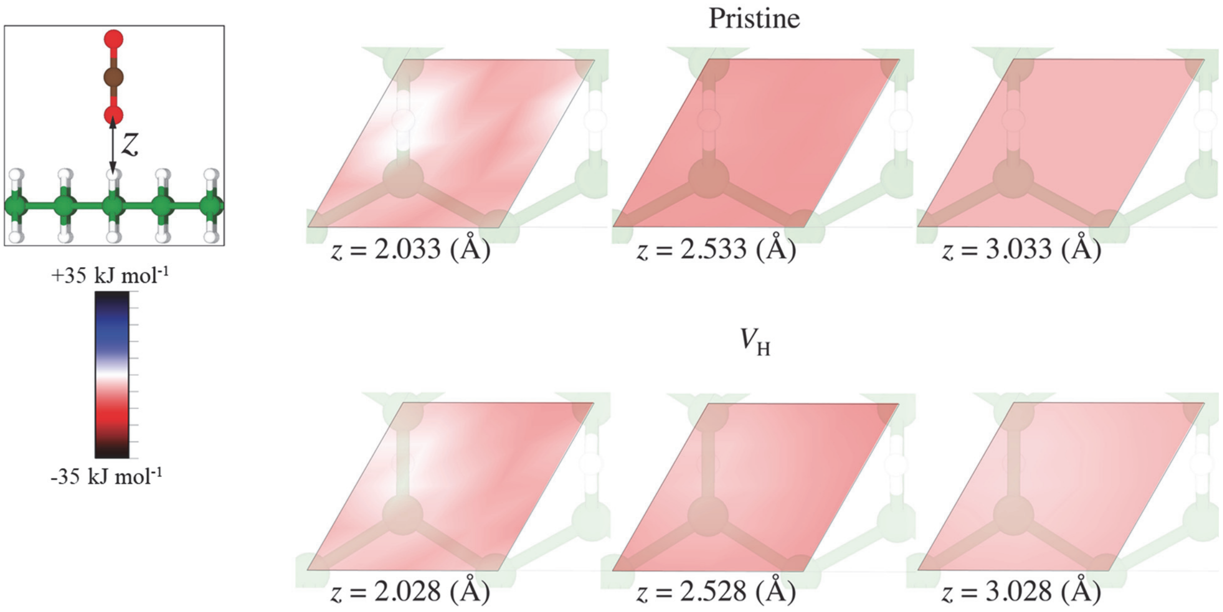

Supplementary Figure 6 | Potential energy surfaces for CO_2 in the end-on configuration on pristine and H-vacant HB sheets (V_H) at various CO_2–HB sheet distances (*z*). The adsorption configuration is shown on the left: *z* is defined by the difference between the *z*-coordinate of the bottom O atom and the average *z*-coordinate of the surface H atoms on HB. In the calculation, the C atom of CO_2 was placed on a 4 × 4 grid of the primitive surface unit cell of HB. The values of the adsorption energy are shown in the bar on the left, where blue (or red) indicates repulsive (or attractive) interaction of CO_2 with the HB sheet. The interaction of CO_2 with the HB sheet is attractive but very weak regardless of the H vacancy, as indicated by the pale red iso-surfaces.

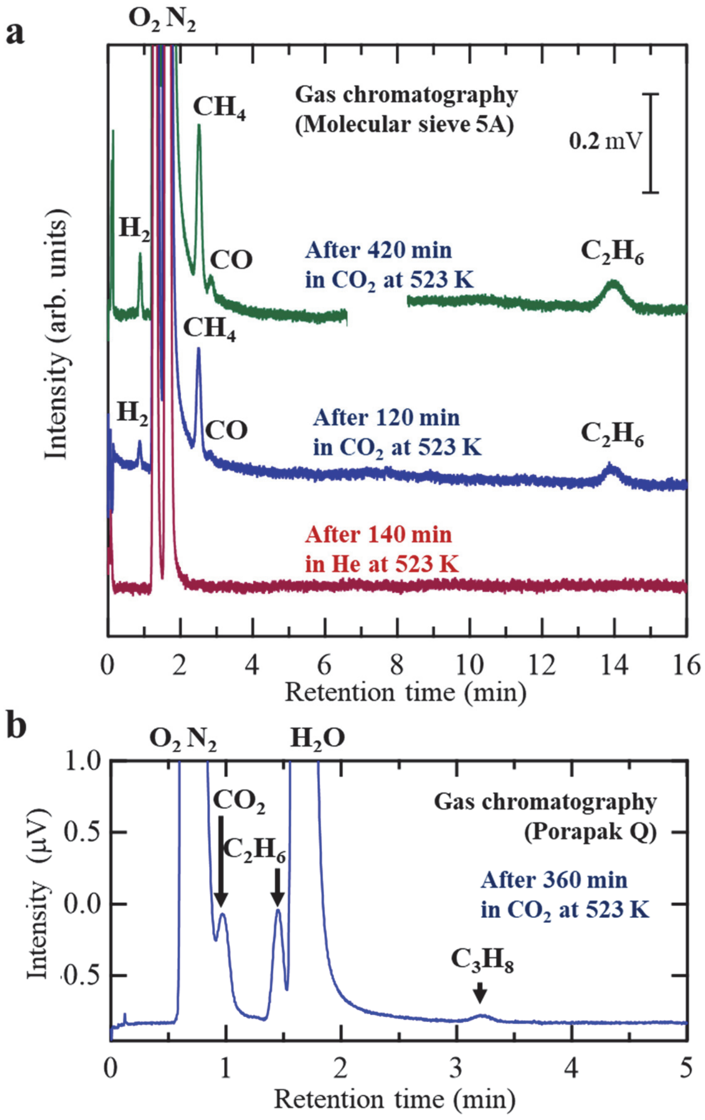

Supplementary Figure 7 | Reaction products of CO_2 (10 cm^3) and HB sheets (100 mg) at 523 K under moist conditions (0.1 cm^3 H_2O). Typical examples of gas chromatographic analysis using **a)** a molecular sieve (5 Å) and **b)** Porapak Q columns. For comparison, the result while using He (no CO_2) is also shown in panel **a**. We note that O_2 and N_2 were detected from the inevitable inclusion of air in the syringe during sampling in every case. However, we found that the O_2/N_2 intensity ratio was larger than that of air in the experiments with CO_2 and H_2O, while the experiment with Ar showed the same O_2/N_2 intensity ratio as that of air. Thus, we estimated the O_2 intensity in the experiments with CO_2 and H_2O by subtracting the background O_2 amount, as estimated from the detected N_2 intensity and the O_2/N_2 ratio of air (measured beforehand). Thus, O_2, CH_4, C_2H_6, and CO were detected when the HB sheets were heated in CO_2 and H_2O (exemplary quantities are shown in **Supplementary Table 1**). We note here that the amount of O_2 dissolved in the inputted H_2O was three orders of magnitude smaller than the detected amount. We sometimes detected small quantities of C_3H_8, as shown in **b**. These signals did not appear upon heating in a He atmosphere; thus, these species were identified as reaction products of HB and CO_2 at 523 K. As such, CO_2 was converted to hydrocarbons, such as methane, ethane, and propane, by reacting with the HB sheets, because CO_2 was the only carbon source in the system.

Supplementary Table 1 | Gas species and amounts detected 350 min after the temperature of the HB sheets in the CO_2 system reached 523 K.

Product	Amount (mol)
O_2	1.1×10^{-5}
CH_4	6.8×10^{-6}
C_2H_6	6.6×10^{-6}
CO	7.3×10^{-7}

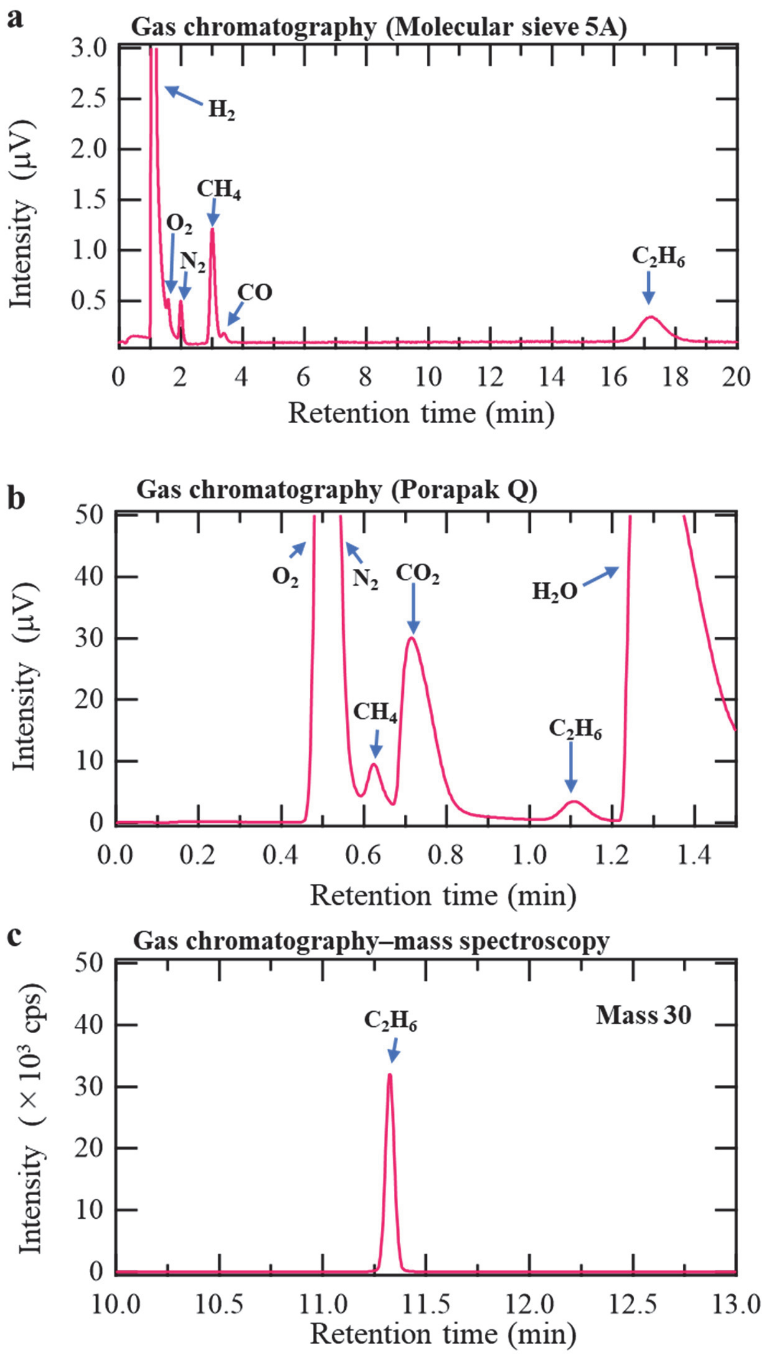

Supplementary Figure 8 | Reaction products of CO_2 (10 cm^3) and HB sheets (100 mg) after 6 h at 523 K under moist conditions (0.1 cm^3 H_2O). Gas chromatography analysis using **a)** a molecular sieve (5 Å) and **b)** Porapak Q columns. **c)** GC-MS analysis at mass number 30 (C_2H_6).

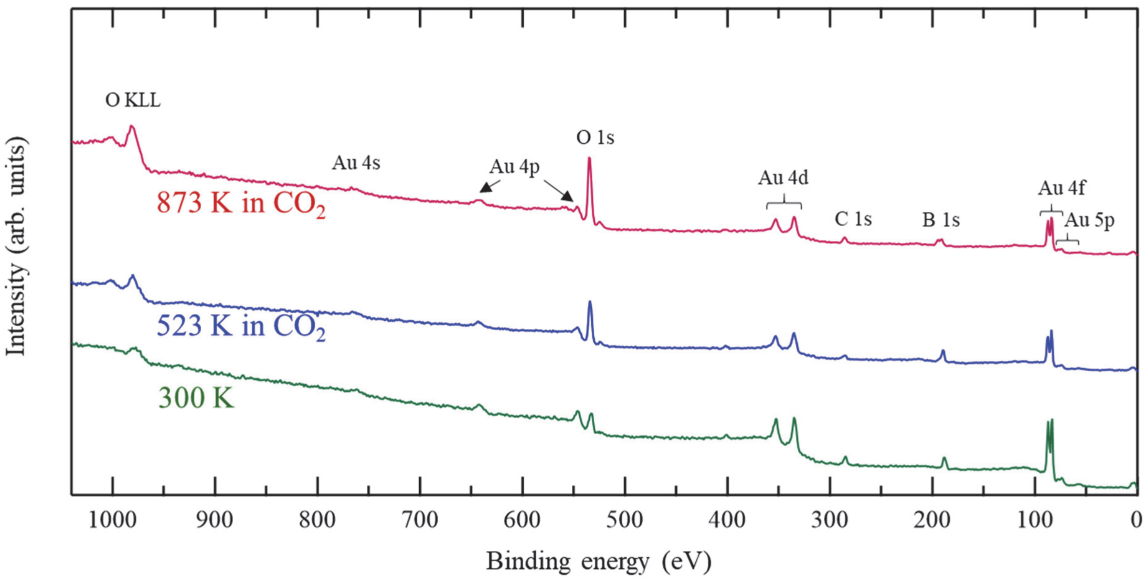

Supplementary Figure 9 | X-ray photoelectron spectroscopy survey scan of HB at 300 K, after heating at 523 K in CO_2, and after heating at 873 K in CO_2. Au sheet was used as the sample holder.

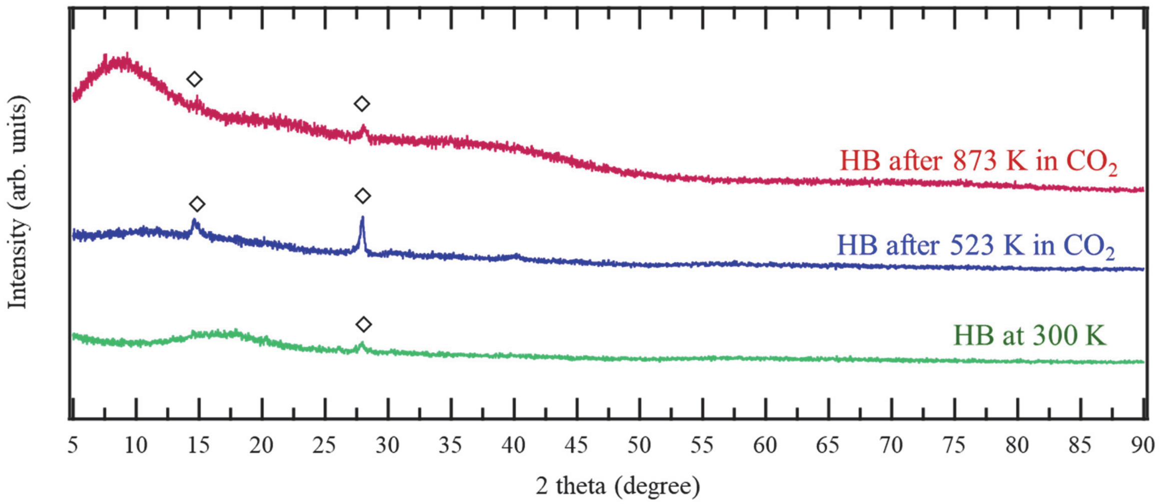

Supplementary Figure 10 | X-ray diffraction (XRD) of HB at 300 K, after heating at 523 K in CO_2, and after heating at 873 K in CO_2. According to the XRD results, the HB sheets exhibited no signs of crystallinity at 300 K, as has been reported previously.[7-11] This property did not change upon increasing the temperature under a CO_2 atmosphere. However, the intensity of the broad peak at approximately 17° weakened at 523 K, and a new broad peak appeared near 8° at 873 K, suggesting continual structural changes for the HB sheets upon heating in CO_2. The peaks at 15° and 28° (indicated by diamond marks) were attributed to the formation of $B(OH)_3$ during the XRD measurements, presumably by reacting with water in ambient air. $B(OH)_3$ is known to decompose above 400 K; thus, these features did not manifest during the heat treatment of the HB sheets in CO_2.
